# WHAT IT MEANS TO ENGAGE IN FALL PREVENTION: QUALITATIVE ANALYSIS OF MOTIVATIONAL INTERVIEWING SESSIONS

**DOI:** 10.1093/geroni/igad104.2079

**Published:** 2023-12-21

**Authors:** Hiroko Kiyoshi-Teo, Siobhan McMahon, Kathlynn Northrup-Snyder

**Affiliations:** Oregon Health & Science University, Portland, Oregon, United States; University of Minnesota, Minneapolis, Minnesota, United States; Consultant, Ilwaco, Washington, United States

## Abstract

Evidence-based strategies to decrease fall risks and rates are well established. However, little is understood about how older people engage in fall prevention strategies. Participants’ statements in the motivational interviewing (MI) sessions conducted as part of Motivational Interviewing for Fall Prevention (MI-FP) study were analyzed. The goal was to describe what it means for older adults to engage in fall prevention; specifically, what and how they engage. Participants (n = 16) were purposefully selected from the MI-FP study for maximum variation in age, sex, fall risks, and assigned MI specialist. Participants were community-dwelling, ≥ 65 years old, and high fall risk for which they received fall prevention recommendations from their primary care provider. Baseline MI sessions were recorded, transcribed and analyzed using qualitative content analysis by three researchers. We first coded fall prevention strategies that participants described. Second, we analyzed the characteristics of these strategies and identified themes. Third, we examined how participants engaged in these strategies. Theoretical frameworks were not used for analysis; however, we did use the Capability, Opportunity, and Motivation Behavior model to organize findings. We found participants 1) used evidence-based and personal strategies; 2) engaged in multiple, personally meaningful strategies and engaged at intensities that worked for them; and 3) were influenced by factors such as their capability to engage in fall prevention, their motivation to engage, and access to opportunities. This study identified what matters for older people to engage in fall prevention.

